# Marburg Virus Outbreak in Equatorial Guinea: Need for Speed

**DOI:** 10.5334/aogh.4178

**Published:** 2024-01-25

**Authors:** Kengo Nathan Ezie, Berjo Dongmo Takoutsing, Diele Modeste, Miste Zourmba Ines, Tatsadjieu Ngoune Leopoldine Sybile, Nformi Monde Caleb, Ignatius N. Esene

**Affiliations:** 1 Faculty of Medicine and Biomedical Sciences of Garoua, University of Garoua Cameroon, Cameroon; 2 Research Division, Winners Foundation, Yaounde, Cameroon; 3 Research Department, Association of Future African Neurosurgeons, Yaounde, Cameroon; 4 Faculty of Health Science, University of Bamenda, Bambili, Cameroon

**Keywords:** Equatorial Guinea, Disease Outbreaks, Marburg Virus Disease, viral hemorrhagic fever

## Abstract

The co-existence of deadly viral pandemics can be considered a nightmare for public health authorities. The surge of a Marburg virus disease (MVD) outbreak in Africa at a time when the coronavirus-19 (COVID-19) pandemic is partially controlled with its limited resources is an urgent call for concern. Over the past decades, several bouts of MVD outbreaks have occurred in Africa with an alarming case fatality rate. Despite this, little has been done to end its recurrence, and affected countries essentially depend on preventative rather than curative measures of management. The recent outbreak of MVD declared by the health officials of Equatorial Guinea, causing several deaths in the context of the COVID-19 pandemic, signals the need for speed in the establishment and the implementation of appropriate health policies and health system strategies to contain, destroy, and prevent the spread of this deadly virus to other neighboring countries.

## 1 Background

At the time when the coronavirus-19 (COVID-19) pandemic is being controlled, another virus enters the scene, the Marburg virus (MARV). An outbreak of this virus occurred on the African continent, which was already facing difficulties managing the COVID-19 pandemic. The Marburg Virus Disease (MVD), caused by the MARV, is well known for causing hemorrhagic fever in both human and non-human primates [1]. It is of significant interest and cause for concern, as it has a high case fatality rate, which ranges between 23–90% with no available cure [1]. Therefore, there is a need for: speed during early case identification; available, affordable, and effective supportive treatment; and vaccination measures to combat the outbreak.

The MARV is a dangerous relative of the Ebola virus (EBOV), both belonging to the family of filoviridae (filoviruses) [2]. It infects people, causing severe viral hemorrhagic fever (VHF), with a morbidity rate approximating 50%. This gives it a reputation of being the deadliest virus ever discovered [2]. Hemorrhagic fever outbreaks that occurred concurrently in laboratories in Marburg, Frankfurt, and Belgrade, Yugoslavia in 1967 led to the discovery of the Marburg virus, with seven reported deaths. The first infected individuals were said to have come into contact with African green monkeys imported from Uganda or their tissues while conducting research [3]. Since the initial epidemic, the first known MVD outbreak in Africa occurred in South Africa eight years later in February 1975, claiming the lives of three persons [4]. Other outbreaks on the continent have followed in Kenya, the Democratic Republic of the Congo, Angola, Uganda, and Ghana (Table 1) [4].

**Table 1:** Chronological repartition of Marburg virus outbreaks in Africa.


COUNTRY	YEAR(S) OF OUTBREAK	TOTAL NUMBER OF CASES

**South Africa**	1975	3

**Kenya**	1980	2

**Kenya**	1987	1

**Democratic Republic of the Congo**	1998–2000	154

**Angola**	2004–2005	374

	2008	2

**Uganda**	2012	15

	2014	1

	2017	3

**Ghana**	2022	10


Adapted from Wellington et AI. 2023 [4].

In countries where the virus is endemic—such as the sub-Saharan African countries, Germany, and Yugoslavia—the exposure rate is higher. The MVD had its greatest impact on the African continent during its outbreak in Angola in 2004 (Table 1). On February 13, 2023, government representatives in Equatorial Guinea reported a Marburg disease outbreak in the northeastern province of Kie-Ntem, with one confirmed case and additional suspected cases [5]. The recurrent outbreaks of the MVD—which has a fatality rate as high as Ebola (23–90%)—on the African continent, most recently in Guinea, lays a major financial burden on this low-income country. It is the third health crisis of public health interest that has occurred in Guinea in the last five years after the COVID-19 pandemic and the EBOV outbreak in 2021 [2].

## 2 Pathophysiology

In addition to *Hipposideros caffer* and several unidentified Chiroptera, *Rousettus aegyptiacus* is the species of bat that most usually serves as a reservoir of MARV [6]. Research has shown that the lungs, intestines, kidneys, bladders, salivary glands, and female reproductive tracts of infected bats contain MARV. Thus, transmission is said to happen either vertically or horizontally inside reservoirs [7]. The two probable mechanisms of bat-to-bat infection include biting and sexual intercourse with other bats [8]. Humans, on the other hand, are primarily infected by the consumption of tainted food by the saliva, urine, or feces of an infected bat [8]. The consumption of the meat of killed non-human primates is another possible mode of bat-to-human transmission. Bodily fluid exchange also accounts for human-to-human transmission [9].

Upon penetration through skin lesions or mucosal surfaces, dendritic cells, macrophages, liver parenchyma cells, adrenocortical cells, and a variety of lymphoid organs are the main target cells of the MARV. Infection of the dendritic cells “paralyzes” the innate immune system responses necessary to initiate an antiviral state. This increases systemic viral reproduction in vivo, coupled with an impairment in T-lymphocyte activation [9]. The apoptosis of T-cells—an endothelial injury that increases vascular permeability—and the widespread intravascular coagulopathy that follow occur as a result of macrophages releasing tumor necrosis factor-alpha. A decrease in coagulation factors brought on by hepatocyte infection amplifies this even further [6,9].

## 3 Clinical Presentation and Diagnosis

**Figure 1:**
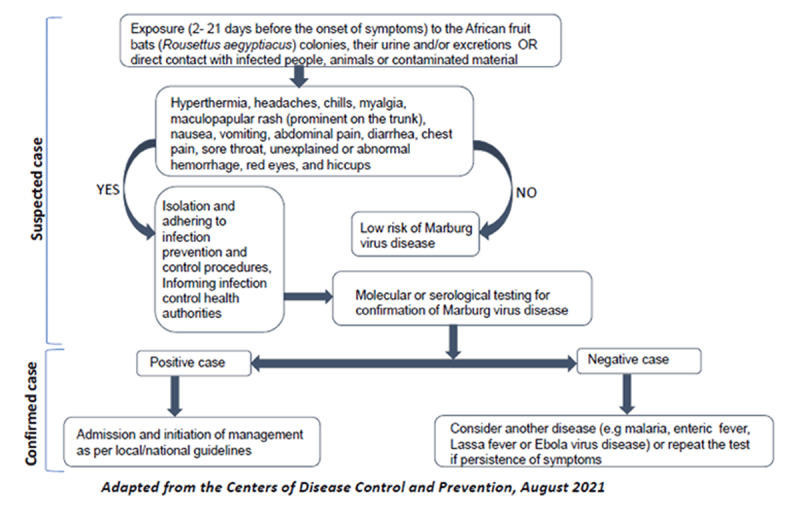
Diagnosis flow chart of Marburg virus disease.

A travel history to endemic zones or exposure, clinical signs, and laboratory testing are necessary to confirm the diagnosis of MVD (Figure 1). Many variables, including the medical environment, host vulnerability, genetics, and viral strain virulence might affect the clinical syndromes brought on by the MARV and the severity of the associated disease [9]. In general, the MARV infection that causes human VHF leads to fluid imbalances, hypotension, coagulopathy, and immunosuppression, culminating in fulminant shock and a multi-organ system failure [10]. According to the course of the illness, VHF has three distinct stages: the generalization phase, the early organ phase, and the late organ phase or convalescence phase [9]. The generalization phase of the illness begins with influenza-like symptoms that last for around five days and includes a high fever (>40°C), chills, myalgia, and malaise after the onset of the sickness [11]. In addition to vomiting, nausea, non-specific discomfort, and appetite loss, severe watery diarrhea, abdominal pain, and fatigue have also been reported [6,11]. Also frequent are pharyngitis, conjunctivitis, and exanthema. At the middle-to-late stage of the generalization phase, a rash may also develop on the face, trunk, and extremities before transforming into a maculopapular rash [6].

The early organ phase, which lasts from days 5 to 13 after the start of the illness, is marked by prostration, dyspnoea, exanthema, and abnormal vascular permeability, including conjunctival injection and edema [9]. Patients may also experience neurological manifestations, such as disorientation, delirium, irritability, aggression, and high temperature [9,11], as well as petechiae, mucosal hemorrhage, uncontrolled bleeding from venipuncture sites, visceral hemorrhagic effusions, hematemesis, and melena, which are symptoms of coagulopathy during this period [9]. When MARV infection reaches a late stage, one of two possibilities occurs: the patient enters a drawn-out phase of recovery, or the infection is deadly [6]. The bulk of deaths in 25 well-documented instances of Marburg and Ebola hemorrhagic fever occurred in the second week of the disease, with a median survival of nine days from the start of the illness until death [12]. In circumstances where the patient survives, the late organ phase begins on day 13 and continues through day 20 and beyond. Severe metabolic abnormalities, convulsions, and extreme dehydration poorly affect the patient’s overall health and can lead to renal failure and multi-organ dysfunction. In certain situations, orchitis and the persistence of neurological symptoms occur during this stage. An additional complication includes a spontaneous abortion in pregnant women [13].

Molecular, serological, and virological techniques are crucial in laboratory diagnosis and differentiate it from other causes of VHF, such as EBOV. Blood (whole blood and serum) is the most acceptable and trustworthy specimen for diagnosis. If blood is not available, other samples such as saliva (oral swab), urine (less reliable), and breast milk can be used as substitute specimen sources [14]. The current methods developed to detect MARV RNA in clinical specimens include standard reverse transcription polymerase chain reaction, reverse transcription loop-mediated isothermal amplification, and quantitative real-time reverse transcription polymerase chain reaction [13,15]. IgM-capture enzyme-linked immunosorbent assays (ELISAs), and direct IgM and IgG ELISAs are frequently employed to detect virus-specific antibodies. IgG antibodies can last several years, though MARV-specific IgM antibodies can emerge as early as two days post-onset of symptoms and decrease between 30 and 168 days after infection. IgG ELISAs are generally used to identify people who have recovered from MHF infection, or for carrying out epidemiological (sero-surveys) and epizoological research. IgM-capture ELISAs on the other hand are more typically employed for the diagnosis of acute sickness [9,15].

## 4 Treatment and Prognosis

There is no standardized Marburg virus disease management strategy. Nonetheless, supportive care is provided, such as blood volume maintenance, electrolyte balance replacing lost blood and clotting factors, and treatment for any complicating infections [16]. Consequently, the outlook for the illness is still grim with a high case fatality rate [9]. Thus, the increasing occurrence of more severe outbreaks suggests that MARV may soon constitute a far greater threat to public health than it presently does. There is a need for speed, as this requires a rapid and effective repost for a better prognosis.

## 5 Current State and Challenges

As declared by Equatorial Guinea’s Health Ministry on February 13, 2023, nine deaths with comparable symptoms associated with VHF occurred between January 7 and February 6, 2023 in two isolated districts of the country’s continental area, Nosrk Nsomo and Ebebiyim [17]. Since the laboratory capacity to test for VHF was unavailable in Equatorial Guinea, ill and close contact samples were transferred to Gabon and Senegal. The samples sent to Gabon for testing came back negative, but one of the eight samples sent to Dakar, Senegal, tested positive [17]. The epidemiological situation as of March 5, 2023 revealed: 9 fatalities (8 likely, 1 confirmed), 16 isolated suspect cases, 2 of which displayed symptoms, and 21 isolated at home, regarded as “secondary contacts”. During this same period, approximately 4,325 persons were under quarantine, and access to Kie Ntem District was restrained [18]. Two other cases were reportedly suspected along the country’s borders with Cameroon, but the country’s health minister denied such claims [19].

This outbreak occurred when the nation lacked the necessary resources to control it, as there is no vaccination or cure for MVD [20]. For the prevention of transmission, Africa’s Centers for Disease Control and Prevention recommend following the same infection prevention and control measures as other VHFs like Ebola [20]. National teams deployed to the afflicted districts had to trace the origin of the outbreak, detect cases, track down contacts, isolate individuals, and treat them. To support national response efforts and guarantee community engagement, the World Health Organization (WHO) deployed experts in epidemiology, case management, infection prevention, laboratory, and risk communication [ref]. In addition, the WHO facilitated the delivery of tents, tools for sample collection and analysis, and a VHF kit with protective gear for 500 medical personnel. Since there were plans to set up laboratory facilities locally, the WHO helped transport samples to labs in Senegal and Gabon [21]. These measures by both national and international organizations placed the country at a sufficient level to manage and contain the outbreak.

Current statistics might not accurately reflect the extent of the outbreak because of the low levels of contact tracing, the small number of samples collected, the lack of testing facilities in the country, the time-lapse for collecting and sending samples to other countries, and delays in receiving results. The aforementioned factors, along with the time elapsed between the discovery of the first symptoms (7 January) and the virus confirmation (13 February), significantly increased the risk of widespread infection in neighboring countries like Cameroon and Gabon. This hinders the initial response and overall management of an extremely virulent outbreak, which precludes the availability of a vaccine, as WHO gives preference to preventive measures such as quarantine [19].

## 6 Strengths and Opportunities

The national training of responders in surveillance, case management, infection prevention and control, safe and respectful burial, risk communication, and community involvement is supported by WHO [17]. Under the leadership of the Minister of Health and the District Chief Medical Officer, the government activated the public health emergency operation center at Ebibeyin. It is currently operational in Bata and aimed at raising awareness and disseminating information on prevention, as WHO supports continuous community engagement initiatives. The existence of a hotline has allowed for the establishment of alert systems [22]. Also, the Centers for Disease Control and Prevention made contact with several non-governmental organizations operating in the impacted regions. It educated these organizations on how to examine the education and health status of staff members before departure, during deployment, and after arrival [23].

## 7 Conclusion and Recommendations

The MARV remains the deadliest virus ever discovered, and its outbreak requires a lot of vigilance and promptness in the initial response. The MVD outbreak, occurring at a time when Equatorial Guinea like other African countries is still recovering from the recent viral pandemic, is no good sign for the nation nor the international community, as adequate response measures are limited. It is thus imperative for the application of effective preventive measures and the rapid installation of detection facilities in the affected areas. The necessity of vaccines and specific viral treatment is not to be argued, as it would be very crucial in contending with the virus and prohibiting further spread. The affected community is called upon to be very vigilant and report any possible cases, while respecting all necessary preventive measures. The country is equally required to carry out high sensitization measures in affected localities to prevent continuous dissemination. This also concerns neighboring countries due to the high level of trade and transborder movements. Putting in place surveillance measures, implementing prevention measures, and anticipating testing kits is the best option if an outbreak were ever to surface. With this, there is a need for speed in reaction to suspected cases.
